# Ovarian SUMO-2/3 targets and their differential response to genotoxic stress induced by 7,12-dimethylbenz(a) anthracene exposure in lean and obese female mice^†^

**DOI:** 10.1093/biolre/ioaf101

**Published:** 2025-04-30

**Authors:** Jaspreet K Rishi, Christian Montes, Justin W Walley, Aileen F Keating

**Affiliations:** Department of Animal Science, Iowa State University, 50011 Ames, IA, USA; Department of Plant Pathology, Entomology and Microbiology, Iowa State University, 50011 Ames, IA, USA; Department of Plant Pathology, Entomology and Microbiology, Iowa State University, 50011 Ames, IA, USA; Department of Animal Science, Iowa State University, 50011 Ames, IA, USA

**Keywords:** SUMOylation, SUMO-2/3, ovary, obesity, DMBA

## Abstract

SUMOylation is a post-translational modification critical for oocyte development and chromatin-associated processes. Environmental exposures and obesity can cause follicle depletion and exposure to 7,12-dimethylbenz(a)anthracene (DMBA) altered ovarian *s*mall *u*biquitin-like *mo*difiers (SUMO) protein abundance in lean and obese mice. Thus, the hypothesis that exposure to DMBA may alter ovarian protein SUMOylation as a mode of ovotoxicity dampened by obesity was tested. Lean and obese mice (KK.Cg-a/a and KK.Cg-Ay/J) were exposed to either corn oil or DMBA (1 mg/kg) intraperitoneally for 7d (n = 4/treatment) and ovaries were flash-frozen on the second day of diestrus. Protein was isolated followed by immunoprecipitation using a SUMO-2/3 antibody and precipitated proteins were identified via liquid chromatography–tandem mass spectrometry. A total of 114 SUMO-2/3 ovarian protein targets were identified. Obesity basally altered (*P* ≤ 0.05) the abundance of 55 SUMOylated proteins with an additional 11 proteins tending (*P* ≤ 0.1) to be altered. In lean mice, DMBA altered (*P* < 0.05) the level of SUMOylation profile of 18 proteins with an additional three proteins tending (*P* ≤ 0.1) to be changed by DMBA exposure. In obese mice, DMBA exposure altered (*P* < 0.05) the abundance of 29 SUMOylated proteins and seven proteins had a tendency toward being different (*P* ≤ 0.1). DMBA exposure of lean compared to obese mice affected (*P* < 0.05) SUMOylation of 43 proteins, and four additional proteins tended (*P* ≤ 0.1) to differ. These findings suggest that protein SUMOylation is a mode of ovotoxicity which is influenced by physiological status.

## Teaser

Obesity may perturb ovarian homeostasis by modulating SUMOylation of ovarian proteins and 7,12-dimethylbenz(a)anthracene exposure selectively affects protein SUMOylation.

## Graphical Abstract

**Graphical Abstract:**
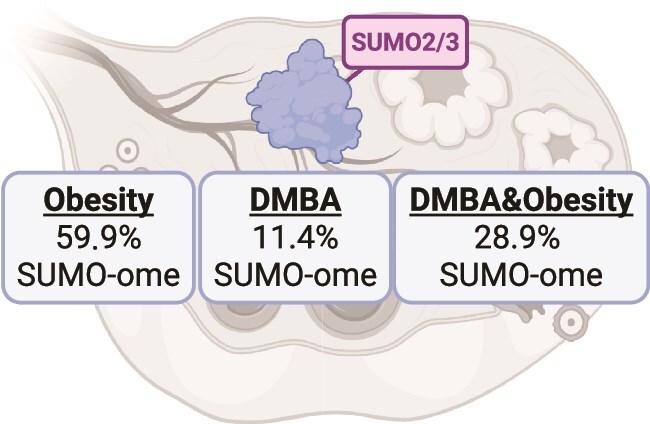

## Introduction

Ovarian function is crucial for female reproductive and general health with the functional follicle unit composed of the germ cell (oocyte) surrounded by somatic cells (granulosa and/or theca cells) [1]. Although most follicles undergo atresia, a subset mature toward ovulation [1, 2]. Exhaustion of the follicular pool results in ovarian senescence termed menopause in women [1]. Premature ovarian follicle depletion leads to premature ovarian insufficiency and is associated with long-term health impairments for women [3–6]. Primary ovarian insufficiency (POI) before age 40 affects ⁓1% of women in the United States and 3.7% of women worldwide [7, 8]. In 90% of cases, POI is idiopathic [9], but environmental exposures contribute by accelerating follicle depletion [10–13]. Women with premature menopause are reported to have a higher incidence of obesity [14], and obesity itself can disrupt the ovarian response to ovotoxic environmental exposures [15–19].

Post-translational modifications (PTM) can modulate histone and non-histone protein activity [20] and include acetylation, glycation, glycosylation, glutathionylation, hydroxylation, lipidation, methylation, *S*-nitrosylation, succinylation, phosphorylation, ubiquitination and SUMOylation [21]. In female reproduction, PTM are involved in the DNA damage response, chromosome condensation and cytoskeletal organization during oocyte maturation [22]. However, epigenetic regulation related to ovarian toxicology is underexplored.

**Figure 1:**
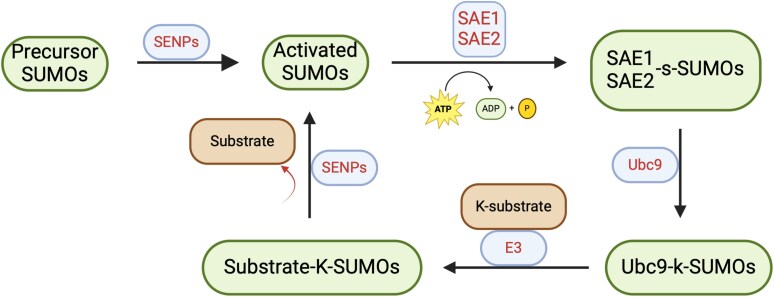
Overview of the cellular SUMO pathway. Newly translated SUMO precursors are cleaved at the C-terminus by SENPs into a mature form to reveal C-terminal di-glycine motifs which form a thioester bond with SUMO-activating enzyme (SAE1 and SAE2) in the presence of ATP. The thioester linkage is transferred to the E2/conjugating enzyme, UBC9. UBC9 conjugates SUMO protein to the Lys residues of a substrate through the action of SUMO E3 ligase to form an isopeptide bond. The process of SUMOylation can be reversed by SENPs cleaving the SUMO terminal glycine from the lysine residues of the substrates, revealing the nascent protein.

Mammalian cells express three to four types of *s*mall *u*biquitin-like *mo*difiers (SUMO) proteins [23] that are involved in SUMOylation [23–25]. Proteins modified by SUMOylation have roles in DNA repair [26–29], transcription [30], nuclear organization [27, 31] and mitosis [32]. SUMOylation can occur both in the cytoplasm and the nucleus on protein lysine residues and the SUMO conjugation machinery is highly conserved in eukaryotes [33, 34]. SUMOylation is reversible by the action of ubiquitin-like protein-specific proteases (ULP) and centrin-specific proteases (SENP) [35, 36]. Newly translated SUMO precursors are processed by SENPs to reveal C-terminal di-glycine motifs, which form a thioester bond with SUMO activating enzymes (SAE1 and SAE2). The thioester linkage is transferred to the E2/conjugating enzyme, ubC9 which conjugates SUMOs onto substrates through an isopeptide bond and the conjugation is facilitated by E3 [37]. Akin to dephosphorylation, SENP and ULP proteins can remove SUMO from the conjugated species to reveal the nascent protein, referred to as deSUMOylation [35] (Figure 1).

In mice, all three SUMO homologs have been identified during the process of oogenesis [38]. SUMO-2 mutants are embryonically lethal, whereas SUMO-1 and SUMO-3 mutants are viable [39–41]. Mutations in SUMO proteases also impair embryo development or result in lethality [42–45]. SUMOylation is therefore seemingly essential for oocyte development and functions in meiotic resumption, meiotic progression and spindle formation during oocyte maturation [46, 47]. Loss of UBC9, an E2 SUMO-conjugating enzyme, causes infertility in mice [48] while loss of UBE2I impaired fertility and affected abundance of genes involved in embryonic development [49]. Thus, SUMOylation is a PTM that has impacts on female fertility.

The ovarian DNA damage repair response (DDR) includes non-homologous end joining (NHEJ) in somatic cells, and homologous recombination (HR) in oocytes [50]. Exposure to the model genetic and ovarian toxicant, 7,12-dimethylbenz(a)anthracene (DMBA) caused follicle loss [51], changed the abundance of ovarian SUMO proteins and this SUMO response differed between lean and obese mice [52]. In obese mice, DMBA exposure increased all three ovarian SUMO isoform proteins [52].

Accumulation of SUMO-1, SUMO-2 and SUMO-3 has been reported at mammalian DNA double strand break (DSB) sites along with the E3 SUMO ligase enzymes PIAS1 and PIAS4 [53]. Since information on the role of SUMOylation in ovarian function is limited and its potential involvement in regulating the ovarian response to environmental stressors remains largely uninvestigated, this study explored the global changes in the SUMO proteome, the “SUMO-ome” [54], in the ovary in response to DMBA exposure in lean and obese mice. The experimental paradigm mimicked a previous study in which lean or obese mice received either corn oil or DMBA for 7 days and alterations in the abundance of SUMO pathway proteins were observed [52]. The aim was to identify ovarian SUMO-2/3 substrates in response to DMBA and to determine if the SUMO-ome differed between lean and obese mice.

## Methods

### Reagents

DMBA (CAS # 57–97-6), corn oil, dithiothreitol, ethylenediaminetetraacetic acid (EDTA), ethanol, iodoacetamide, Tris HCl Tris Base and trypsin were obtained from Sigma-Aldrich (St. Louis, MO, USA). Tween 20 was obtained from Fisher Bioreagents (Fair Lawn, NH, USA). A Micro-Pillar Array Column (110 cm), High pH Reversed-Phase Peptide Fractionation Kit, PepMap Neo trap column (300 μM i.d. × 5 mm), 5 μm C18, 100 Å μ-Precolumn, reducing loading buffer, and TMTpro were purchased from Thermo Fisher Scientific (Rockfield, IL, USA). The Universal Magnetic Co-IP Kit (catalog # 54002) was purchased from Active Motif (Carlsbad, CA, USA). SUMO2/3 polyclonal antibody (catalog # 11251-1AP) was obtained from Proteintech (Rosemont, IL, USA). Liquid nitrogen was from Iowa State University Chemistry store.

### Animal exposure and tissue collection

All animal use was approved by the Iowa State University Institutional Animal Care and Use Committee. Female hyperphagic lean (HPL) C57Bl6 (KK.Cg-a/a; strain # 000664; n = 20) and hyperphagic obese (HPO) agouti lethal yellow mice (KK.Cg-Ay/J; strain # 002468; n = 20) were obtained from Jackson Laboratory (Bar Harbor, ME) and housed 2–5 per cage. The mice were maintained on a 12-h light/dark cycle with the room temperature at 25°C. The animals had *ad libitum* access to food (2014 Envigo Teklad Global 14% Protein Rodent Maintenance Diet) and water, and weekly food intake and body weights were monitored. Once the HPL and HPO mice had a ~ 30% weight differential (~9 weeks old), they received either corn oil as vehicle control (CT) or DMBA (1 mg/Kg/day) via intraperitoneal injection. The DMBA dose, route of exposure and duration were based upon previous work establishing this paradigm as causing ovotoxicity and altering SUMO proteins [51, 52]. The stage of the estrous cycle was monitored daily via vaginal cytological analysis, and euthanasia occurred at ~10 wk of age on the second day of diestrus following dosing. Total body weight, along with the weights of spleen, liver, ovary and uterus were recorded. Blood was collected immediately via cardiac puncture, and serum was collected and stored at −80°C. Ovaries from each mouse were frozen in liquid nitrogen and stored at −80°C.

### Protein isolation and co-immunoprecipitation

Ovarian tissue (n = 4/treatment) was lysed in a buffer containing 50 mM Tris–HCl and 1 mM EDTA by sonication for 30 s and incubation for 30 min. The lysate was centrifuged twice at 10,000 rpm for 15 min and the supernatant quantified using the BCA assay. Whole-cell extract (300 mg) and SUMO2/3 antibody (3 mg) were combined in a final volume of 500 mL of complete co-IP wash buffer on ice and incubated on a rotator at 4°C for 4 h, followed by centrifugation at 4000 rpm for 30 s. Samples were incubated with Protein G Magnetic Beads (25 mL) for 1 h followed by centrifugation at 4000 rpm for 30 s. Magnetic beads were then washed four times using the wash buffer and a magnetic stand was used to pellet the beads, discarding the supernatant. Bead pellets were resuspended in 20 mL of 2× reducing loading buffer. Enriched samples were eluted from beads by incubation at 95°C for 10 min in 1× S-Trap lysis buffer (5% SDS, 50 mM TEAB, pH 8.5) supplemented with 12.5 mM biotin. Eluted samples were subjected to S-Trap sample processing technology. Samples were reduced in 2 mM TCEP, alkylated in 50 mM iodoacetamide, and digested into peptides with an overnight incubation at 37°C with 50 ng of Trypsin and 50 ng of Lys-C. Peptides were further desalted using SepPack C18 columns (Waters). Tandem Mass Tag Pro labeling was performed on purified peptides from each sample and samples were then pooled. TMT-labeled, pooled samples were subjected to high pH fractionation using Pierce High pH Reversed-Phase Peptide Fractionation Kit following manufacturer instructions. The obtained 8 fractions were further pooled as follows: fraction 1 with fraction 5, fraction 2 with fraction 6, fraction 3 with fraction 7, and fraction 4 with 8. Samples were finally dried on a SpeedVac and resuspended in 0.1% Optima™ grade formic acid (Fisher, cat No. A117–50) in Optima™ grade H_2_O (Fisher, car No. W64). An injection volume of 20 μL, containing 1.2 μg from each fraction, was used for LC–MS/MS analysis.

### LC–MS/MS and proteomic analysis

Chromatography was performed on a Thermo Vanquish Neo UHPLC in “heated trap-and-elute, backward flush” mode. Peptides were desalted and concentrated on a PepMap Neo trap column (300 μM i.d. × 5 mm, 5 μm C18, 100 Å μ-Precolumn) at a flow rate of 10 μL/min. Sample separation was performed on a 110 cm Micro-Pillar Array Column with a flow rate of ~300 nL min^−1^ over a 120 min reverse phase active gradient (80% ACN in 0.1% FA from 15% to 43.8% over 109 min, from 43.8% to 62.5% for 11 min) followed by a column/trap wash at 80% ACN for 10 min. Eluted peptides were analyzed using a Thermo Scientific Orbitrap Exploris 480 mass spectrometer with a FAIMS pro Duo interface installed, which was directly coupled to the UHPLC through an Easy Spray Ion source. Data dependent acquisition was obtained using Xcalibur 4.0 software in positive ion mode with a spray voltage of 2.2 kV, a capillary temperature of 280°C, an RF of 45, FAIMS compensation voltages of −45, and −60, and a total carrier gas flow of 4.6 l/min. MS1 spectra were measured at a resolution of 120 000, an automatic gain control (AGC) of 3e6 with auto maximum ion time, and a mass range of 400–1400 m/z. A cycle time of 0.8 s was used to capture triggered MS2 at a resolution of 15 000 with the “Turbo TMTpro” setting on. Fixed first mass of 110 m/z, AGC of 1.0^5^ with a maximum ion time of 22 ms, normalized collision energy of 33, and isolation window of 0.7 m/z were used. Charge inclusion was set to 2–6. MS1 that triggered MS2 scans were dynamically excluded for 30 s.

**Figure 2:**
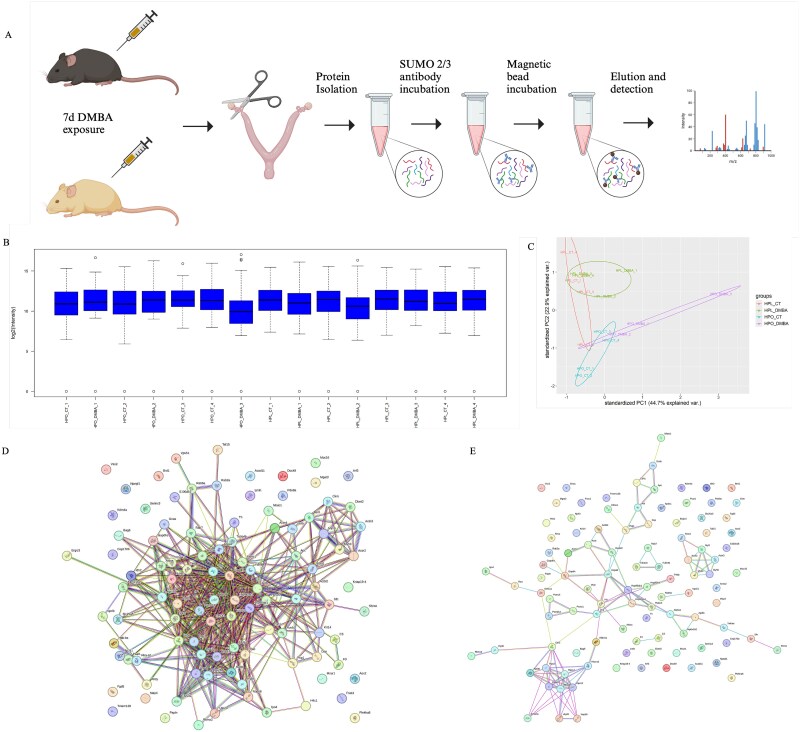
Ovarian SUMOylation-induced protein changes in response to DMBA exposure in lean and obese mice. (A) Schematic of the study design to study the SUMO-ome in response to DMBA exposure in lean and hyperphagia-induced obese mice. (B) Box and whisker plot showing protein intensity distribution for each sample. Each whisker represents the 25% highest and lowest intensity proteins (first and third quartile, respectively). Box represents where 50% of the protein intensity lays. Black center line shows location of intensity median. Extreme values, as defined by the interquartile range criterion, are depicted as open circles. (C) PCA plot. Network of (D) functional and (E) physical interactions of detected proteins. *n* = 4 for HPL-CT, HPL-DMBA and HPO-CT; *n* = 3 for HPO-DMBA.

### Data analysis

Raw data were analyzed using MaxQuant version 2.4.10. Spectra were searched, using the Andromeda search engine, against the *Mus musculus* UNIPROT reference annotation. The proteome files were complemented with reverse decoy sequences and common contaminants by MaxQuant. Carbamidomethyl cysteine was set as a fixed modification while methionine oxidation and protein N-terminal acetylation were set as variable modifications. The sample type was set to “FAIMS-Reporter Ion MS2” with “TMT18plex” selected for both lysine and N-termini. TMT batch-specific correction factors were configured in the MaxQuant modifications tab (TMT Lot No. XA338617). Digestion parameters were set to “specific” and “Trypsin/P;LysC”. Up to two missed cleavages were allowed. False discovery rate was calculated in MaxQuant using a target-decoy strategy. Significant interactors were assessed using the TMT-NEAT R pipeline with the PoissonSeq R package. Proteins that differed at *P* ≤ 0.1 were included for analysis. Altered proteins across treatment groups were compared with the reference database of *M. musculus* on PANTHER v16.0 to visualize gene ontology and protein classes. STRING v12.0 database was used to visualize predicted functional and physical protein–protein interactions. The remaining comparisons and visualizations were performed on GraphPad Prism 9.0. A *P* value <0.05 was considered a significant statistical difference and *P* value ≤0.1 denoting a statistical tendency for biological meaning.

## Results

### The ovarian SUMO-ome has a high functional and physical interaction

**Figure 3:**
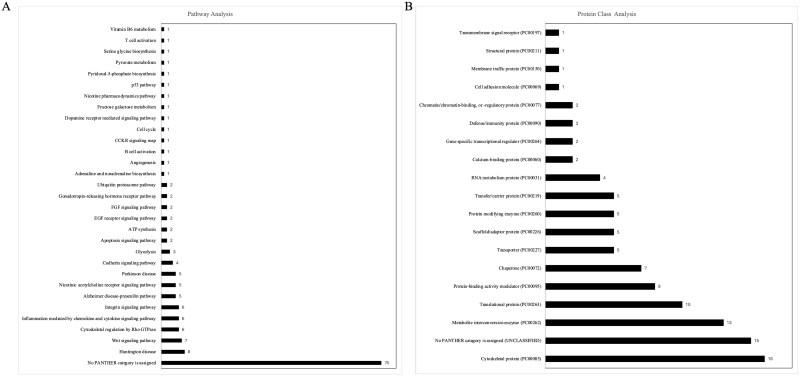
Classification of ovarian proteins SUMOylated in response to DMBA exposure in lean and obese mice. Classification of detected proteins in (A) pathways and (B) protein class. *n* = 4 for HPL-CT, HPL-DMBA and HPO-CT; *n* = 3 for HPO-DMBA.

Phenotypic and estrous cyclicity data for the lean and obese mice is provided in Supplemental Figure 1 and illustrate that obese mice had heavier body and liver weight in agreement with previous findings with this animal experimental design [53]. The protein complexes of interest were immunoprecipitated using the SUMO-2/3 antibody on magnetic beads and eluted protein complexes from immunoprecipitation were analyzed using mass spectrometry (Figure 2A). Box plot analysis demonstrated that peptides from one of the biological replicates (HPO-DMBA # 3) distributed unusually highly toward zero and this sample was omitted from further analysis which did not affect principal component analysis distribution (Figure 2B and C). This approach identified 114 SUMOylated proteins as components of the ovarian SUMO-ome (Table 1). The functional interactions of the detected proteins were assessed by STRING Network Analysis using the STRING database and 103 nodes, while 523 edges were detected (Figure 2D, Supplemental Table 1). Physical interaction network analysis identified 103 nodes and 113 edges (Figure 2E, Supplemental Table 2) and the low protein–protein interaction (PPI) enrichment values highlight the significance of the interactions. Pathway analysis of the “SUMO-ome” using the PANTHER database identified 30 pathways (Figure 3A). Approximately half of the detected proteins were not assigned to a PANTHER category. The highest number of proteins classified were in the Huntington disease category (eight proteins), followed by the WNT signaling pathway (seven proteins). Other pathways of interest detected were the gonadotropin-releasing hormone receptor signaling pathway and the P53 pathway. Classification of the SUMO-ome into protein class identified 18 classes (Figure 3B) and with most proteins being allocated in the cytoskeletal protein category (16 proteins) followed by metabolite interconversion enzymes (13 proteins) and translational proteins (10 proteins).

**Table 1:** SUMOylated proteins detected via LC–MS after co-immunoprecipitation with SUMO-2/3 antibody. *indicates proteins that were also detected in the total ovarian proteome [52].

UNIPROT ID	Protein Name	Protein Symbol
P62259*	14–3-3 protein epsilon	YWHAE
O70456*	14–3-3 protein sigma	SFN
O35593	26S proteasome non-ATPase regulatory subunit 14	PSMD14
P62192	26S proteasome regulatory subunit 4	PSMC1
P62196	26S proteasome regulatory subunit 8	PSMC5
P68134	Actin, alpha skeletal muscle	ACTA1
P60710	Actin, cytoplamethyl smic 1	ACTB
Q80XL6	Acyl-CoA dehydrogenase family member 11	ACAD11
Q61315	Adenomatous polyposis coli protein	APC
P50247*	Adenosylhomocysteinase	AHCY
P61205*	ADP-ribosylation factor 3	ARF3
P51881*	ADP/ATP translocase 2	SLC25A5
Q5SGK3	Aldehyde oxidase 2	AOX2
Q9JI91	Alpha-actinin-2	ACTN2
O88990	Alpha-actinin-3	ACTN3
P57780	Alpha-actinin-4	ACTN4
P07356*	Annexin A2	ANXA2
Q9DCR2	AP-3 complex subunit sigma-1	AP3S1
Q03265*	ATP synthase subunit alpha, mitochondrial	ATP5F1A
P56480*	ATP synthase subunit beta, mitochondrial	ATP5F1B
Q8BFZ3	Beta-actin-like protein 2	ACTBL2
A8DUK4*	Beta-globin	HBB-BS
G5E8P1	Bromodomain-containing protein 1	BRD1
P09803	Cadherin-1	CDH1
Q80U49	Centrosomal protein of 170 kDa protein B	CEP170B
P01027*	Complement C3	C3
P07310	Creatine kinase M-type	CKM
Q6P8J7	Creatine kinase S-type, mitochondrial	CKMT2
Q8BIK4	Dedicator of cytokinesis protein 9	DOCK9
E9Q557	Desmoplakin	DSP
P10126*	Elongation factor 1-alpha 1	EEF1A1
P58252	Elongation factor 2	EEF2
Q9CQE7	Endoplasmic reticulum-Golgi intermediate compartment protein 3	ERGIC3
P08113	Endoplasmin	HSP90B1
P60843	Eukaryotic initiation factor 4A-I	EIF4A1
Q9Z0N1	Eukaryotic translation initiation factor 2 subunit 3, X-linked	EIF2S3X
Q80T14	Extracellular matrix organizing protein FRAS1	FRAS1
Q505K2	FHF complex subunit HOOK-interacting protein 1A	FHIP1A
P05064*	Fructose-bisphosphate aldolase A	ALDOA
E9QLU9	FYVE, RhoGEF and PH domain containing 5	FGD5
Q8VIJ8	GATOR1 complex protein NPRL3	NPRL3
P16858	Glyceraldehyde-3-phosphate dehydrogenase	GAPDH
Q64467	Glyceraldehyde-3-phosphate dehydrogenase, testis-specific	GAPDHS
P62827	GTP-binding nuclear protein Ran	RAN
P16627	Heat shock 70 kDa protein 1-like	HSPA1L
P63017	Heat shock cognate 71 kDa protein	HSPA8
P07901*	Heat shock protein HSP 90-alpha	HSP90AA1
P11499*	Heat shock protein HSP 90-beta	HSP90AB1
P01942	Hemoglobin subunit alpha	HBA
O88502	High affinity cAMP-specific and IBMX-insensitive 3′,5′-cyclic phosphodiesterase 8A	PDE8A
P62806*	Histone H4	H4C1
P42859	Huntingtin	HTT
P01865	Ig gamma-2A chain C region, membrane-bound form	IGH-1A
A0A075B5P4	Immunoglobulin heavy constant gamma 1 (G1m marker)	IGHG1
A0A075B5P3	Immunoglobulin heavy constant gamma 2B	IGHG2B
A0A075B5P5	Immunoglobulin heavy constant gamma 3	IGHG3
A0A0A0MQC1	Immunoglobulin heavy variable 3–5	IGHV3–5
A0A075B5V6	Immunoglobulin heavy variable V1–42	IGHV1–42
A0A140T8M4	Immunoglobulin kappa variable 8–19	IGKV8–19
Q8VI75	Importin-4	IPO4
P54071*	Isocitrate dehydrogenase [NADP], mitochondrial	IDH2
Q02257	Junction plakoglobin	JUP
Q925H7	Keratin-associated protein 19–4	KRTAP19–4
Q61781*	Keratin, type I cytoskeletal 14	KRT14
Q9Z1R2	Large proline-rich protein BAG6	BAG6
P62889*	Large ribosomal subunit protein eL30	RPL30
Q8BMN4	Leishmanolysin-like peptidase	LMLN
Q8BW72	Lysine-specific demethylase 4A	KDM4A
Q5RKT9	Mannoside acetylglucosaminyltransferase 3	MGAT3
P27546*	Microtubule-associated protein 4	MAP4
Q9CXD6	Mitochondrial calcium uniporter regulator 1	MCUR1
A0A140LJ72	Mucin 16	MUC16
P05977	Myosin light chain 1/3, skeletal muscle isoform	MYL1
P97457	Myosin regulatory light chain 11	MYL11
P13541	Myosin-3	MYH3
Q02566	Myosin-6	MYH6
O88942	Nuclear factor of activated T-cells, cytoplasmic 1	NFATC1
Q9D6Z1	Nucleolar protein 56	NOP56
Q3V2K7	Nucleoporin 50 like	NUP50L
A0A286YDM5	Olfactory receptor	OR
Q9EPX2	Papilin	PAPLN
Q99K85	Phosphoserine aminotransferase	PSAT1
P97350	Plakophilin-1	PKP1
Q80W71	Pleckstrin homology domain-containing family A member 8	PLEKHA8
Q99PV0	Pre-mRNA-processing-splicing factor 8	PRPF8
Q6NSR8*	Probable aminopeptidase NPEPL1	NPEPL1
Q6R0H6	Protein ALEX	GNAS
P09103*	Protein disulfide-isomerase	P4HB
Q9D708	Protein S100-A16	S100A16
Q80U72	Protein scribble homolog	SCRIB
P52480*	Pyruvate kinase PKM	PKM
P53994	Ras-related protein Rab-2A	RAB2A
P35279	Ras-related protein Rab-6A	RAB6A
P51150	Ras-related protein Rab-7a	RAB7A
Q8R0F5	RNA-binding motif protein, X-linked 2	RBMX2
Q9WTM5	RuvB-like 2	RUVBL2
Q9QZI9	Serine incorporator 3	SERINC3
Q921I1*	Serotransferrin	TF
G3UXH3	Shroom family member 3	SHROOM3
P62858	Small ribosomal subunit protein eS28	RPS28
P62242*	Small ribosomal subunit protein eS8	RPS8
P62264	Small ribosomal subunit protein uS11	RPS14
P62301	Small ribosomal subunit protein uS15	RPS13
P62908	Small ribosomal subunit protein uS3	RPS3
Q5XG71	Small subunit processome component 20 homolog	UTP20
Q8BQ46*	TAF15 RNA polymerase II, TATA box binding protein (TBP)-associated factor	TAF15
Q01853*	Transitional endoplasmic reticulum ATPase	VCP
Q9CZB9	Transmembrane protein 128	TMEM128
P05213*	Tubulin alpha-1B chain	TUBA1B
P68372*	Tubulin beta-4B chain	TUBB4B
P24529	Tyrosine 3-monooxygenase	TH
B1ARW8	Uncharacterized protein C1orf122 homolog	
P62814*	V-type proton ATPase subunit B, brain isoform	ATP6V1B2
Q3UVL4	Vacuolar protein sorting-associated protein 51 homolog	VPS51

### Ovarian SUMO-ome effect of obesity alone and DMBA exposure in lean and obese mice

**Figure 4:**
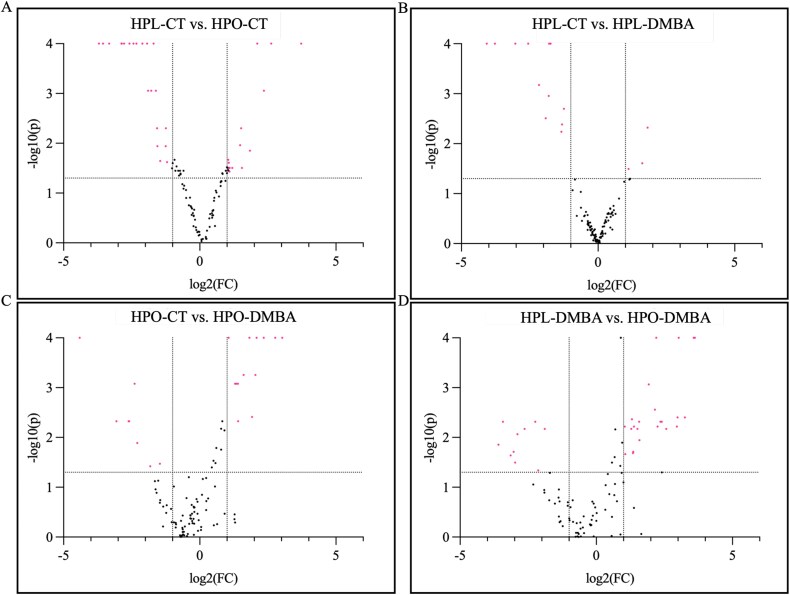
Volcano plot illustrating ovarian proteins SUMOylated in response to DMBA exposure in lean and obese mice. Differential SUMOylation of ovarian proteins is presented in the treatment comparisons, (A) HPL-CT vs. HPL-DMBA, (B) HPL-CT vs. HPO-CT, (C) HPO-CT vs. HPO-DMBA, (D) HPL-DMBA vs. HPO-DMBA. The dotted horizontal line indicates *P* = 0.05, and the dotted vertical lines indicate fold change = ± 2. *n* = 4 for HPL-CT, HPL-DMBA and HPO-CT; *n* = 3 for HPO-DMBA.

**Figure 5:**
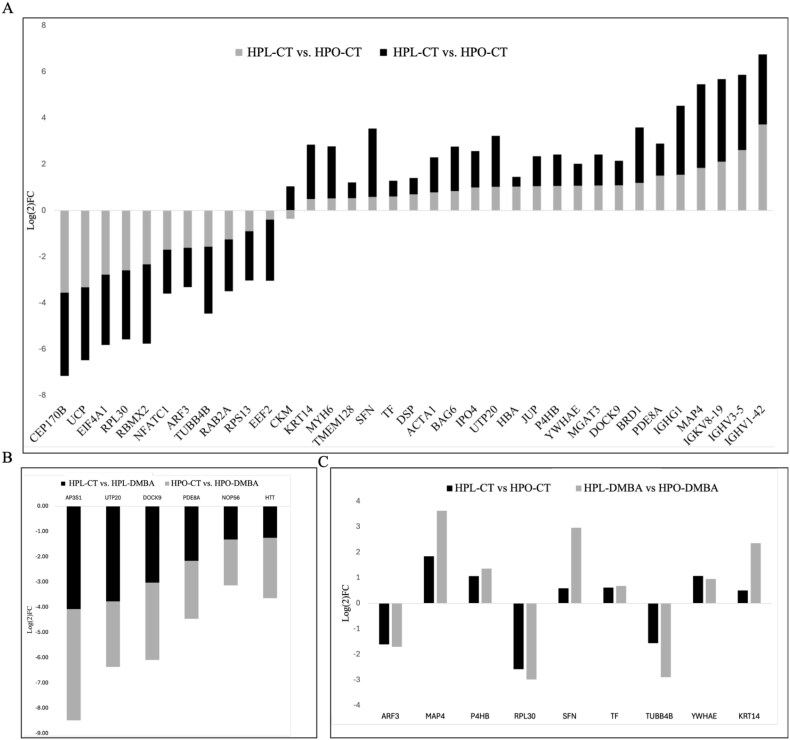
Ovarian SUMOylation substrates identified during obesity and DMBA exposure. (A) Proteins SUMOylated by SUMO-2/3 and affected by obesity regardless of DMBA exposure. (B) Proteins SUMOylated by SUMO-2/3 and affected by DMBA regardless of metabolic status. (C) Proteins SUMOylated by SUMO-2/3 and affected by obesity regardless of DMBA exposure and detected in whole ovary proteomics. *P* = 0.1; *n* = 4 for HPL-CT, HPL-DMBA and HPO-CT; *n* = 3 for HPO-DMBA.

Comparison of the ovarian SUMO-ome between control-treated lean and obese mice (HPL-CT vs HPO-CT) identified 55 proteins as altered (*P* < 0.05) due to obesity and an additional 11 proteins had a tendency (*P* ≤ 0.1) to be changed by obesity (Figure 4A; Supplemental Table 3). In lean mice, exposure to DMBA (HPL-CT vs HPL-DMBA) resulted in 18 SUMOylated proteins being identified (*P* < 0.05), and three tended (*P* ≤ 0.1) to differ (Figure 4B; Supplemental Table 4). In obese mice, DMBA exposure altered the SUMOylation profile of 29 proteins (*P* < 0.05) and seven proteins had a tendency (*P* ≤ 0.1) to be altered (Figure 4C; Supplemental Table 5). In the final comparison, DMBA exposure in the lean compared to obese mice altered (*P* < 0.05) the abundance of 43 SUMOylated proteins, and four additional proteins tended (*P* ≤ 0.1) to be changed (Figure 4D; Supplemental Table 6). Obesity tended to impact 35 SUMOylated proteins (*P* ≤ 0.1) regardless of DMBA exposure (Figure 5A, Table 2). Classification of these proteins into biological processes revealed that obesity affected SUMOylation of proteins involved in processes namely “biological process involved in interspecies interaction between organisms” (1.3%), “biological regulation” (9.2%), “cellular process” (27.6%), “developmental process” (5.3%), “homeostatic process” (1.3%), “immune system process” (3.9%), “localization” (7.9%), “metabolic process” (11.8%), “multicellular organismal process” (3.9%), “response to stimuli” (14.5%). Nine of these 35 proteins were also altered in “whole-ovary proteomics” (Figure 5C, Table 2) [52]. Exposure to DMBA tended to decrease the abundance of six SUMOylated proteins, regardless of metabolic status (*P* ≤ 0.1; Figure 5B, Table 3). These proteins could be classified into the following biological processes: “biological regulation” (25%), “cellular process” (12.5%), “localization” (12.5%) and “response to stimulus” (12.5%).

**Table 2:** Effect of obesity on SUMOylation of ovarian proteins independent of DMBA exposure. * indicates proteins that were also identified in the whole ovary proteomic analysis [52].

*UNIPROT ID*	*Protein Name*	*HPL-CT vs HPO-CT*	*HPL-DMBA vs HPO-DMBA*
Q80U49	Centrosomal protein of 170 kDa protein B	−3.56	−3.59
B1ARW8	Uncharacterized protein	−3.33	−3.15
P60843	Eukaryotic initiation factor 4A-I	−2.78	−3.04
P62889*	60S ribosomal protein L30	−2.59	−2.98
Q8R0F5	RNA-binding motif protein, X-linked 2	−2.33	−3.43
O88942	Nuclear factor of activated T-cells, cytoplasmic 1	−1.70	−1.89
P61205*	ADP-ribosylation factor 3	−1.61	−1.70
P68372*	Tubulin beta-4B chain beta-2B chain	−1.57	−2.89
P53994	Ras-related protein Rab-2A	−1.25	−2.24
P62301	40S ribosomal protein S13	−0.89	−2.13
P58252	Elongation factor 2	−0.40	−2.63
P07310	Creatine kinase M-type	−0.36	1.05
Q61781*	Keratin, type I cytoskeletal 14	0.50	2.35
Q02566	Myosin-6	0.52	2.25
Q9CZB9	Transmembrane protein 128	0.53	0.68
O70456*	14–3-3 protein sigma	0.59	2.96
Q921I1*	Serotransferrin	0.61	0.67
E9Q557	Desmoplakin	0.71	0.70
P68134	Actin, alpha skeletal muscle	0.78	1.51
Q9Z1R2	Large proline-rich protein BAG6	0.84	1.93
Q8VI75	Importin-4	1.00	1.58
Q5XG71	Small subunit processome component 20 homolog	1.03	2.20
P01942	Hemoglobin subunit alpha	1.03	0.42
Q02257	Junction plakoglobin	1.06	1.29
P09103*	Protein disulfide-isomerase	1.06	1.36
P62259*	14–3-3 protein epsilon 1	1.07	0.95
Q5RKT9	Beta-1,4-mannosyl-glycoprotein 4-beta-N-acetylglucosaminyltransferase	1.08	1.35
Q8BIK4	Dedicator of cytokinesis protein 9	1.09	1.06
G5E8P1	Bromodomain-containing protein 1	1.20	2.40
O88502	High affinity cAMP-specific and IBMX-insensitive 3,5-cyclic phosphodiesterase 8A	1.51	1.38
A0A075B5P4	Ig gamma-1 chain C region secreted form (Fragment)	1.55	2.98
P27546*	Microtubule-associated protein 4	1.84	3.62
A0A140T8M4	Immunoglobulin kappa variable 8–19	2.11	3.58
A0A0A0MQC1	Immunoglobulin heavy variable 3–5	2.62	3.25
A0A075B5V6	Immunoglobulin heavy variable V1–42	3.73	3.03

**Table 3:** Effect of DMBA on SUMOylation of ovarian proteins regardless of metabolic status.

*UNIPROT ID*	*Protein Name*	*HPL-CT vs HPL DMBA*	*HPO-CT vs HPO DMBA*
Q9DCR2	AP-3 complex subunit sigma-1	−4.08	−4.41
Q5XG71	Small subunit processome component 20 homolog	−3.77	−2.60
Q8BIK4	Dedicator of cytokinesis protein 9	−3.03	−3.06
O88502	High affinity cAMP-specific and IBMX-insensitive 3,5-cyclic phosphodiesterase 8A	−2.16	−2.30
Q9D6Z1	Nucleolar protein 56	−1.32	−1.82
P42859	Huntingtin	−1.25	−2.39

Comparison of SUMO-ome and whole ovary proteome during DMBA exposure in lean and obese mice.

**Figure 6:**
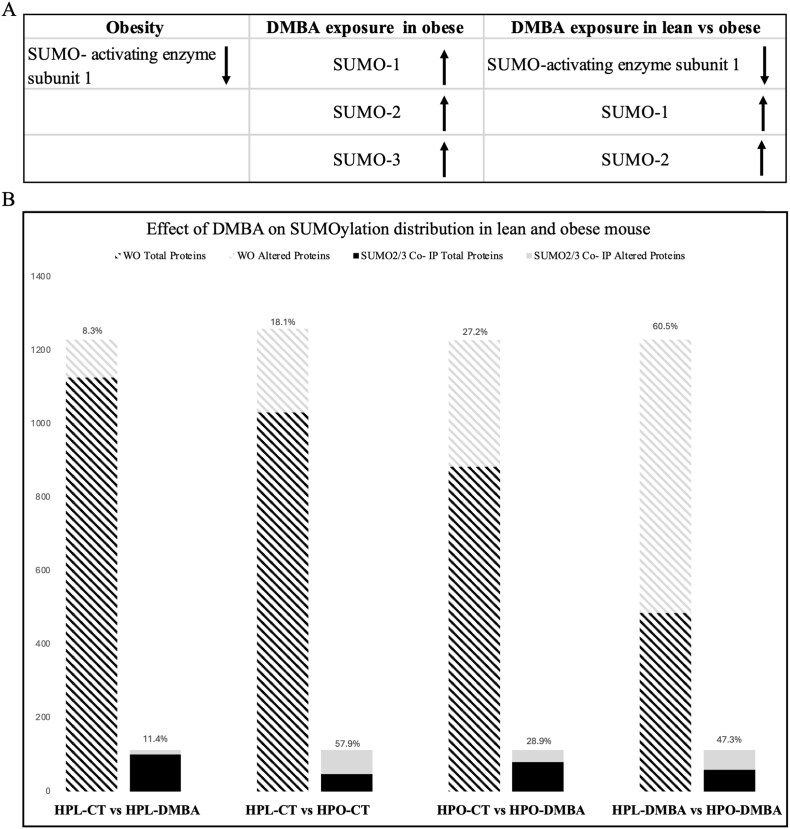
Comparison of whole ovary proteomics and SUMO-2/3 targets. (A) SUMO pathway proteins identified in whole ovary proteome analysis during DMBA exposure in lean and obese mice. (B) Quantitative comparison of the SUMO-ome against the whole ovary proteome. WO = “whole ovary.” *P* = 0.1; *n* = 4 for HPL-CT, HPL-DMBA and HPO-CT; *n* = 3 for HPO-DMBA.

Exposure of lean and obese mice to DMBA elicited a differential ovarian proteomic response [52] with alterations in SUMO pathway proteins observed, while basally obesity tended to increase the abundance of all three SUMO isoforms (*P* ≤ 0.1; Figure 6A). Comparing SUMOylated proteins in the current study with the total ovarian proteome from the previous study [52] revealed that ~9% of the total ovarian proteome underwent SUMOylation. In lean mice, DMBA exposure tended to affect 8.3% of the total ovarian proteome and 11.4% of the SUMO-ome (*P* ≤ 0.1; Figure 6B). In contrast, in obese mice, DMBA exposure tended to affect 27.2% of the total ovarian proteome and 28.9% of the SUMO-ome (*P* ≤ 0.1; Figure 6B). Obesity alone altered 18.1% of the total ovarian proteome and 57.9% of the SUMO-ome (*P* ≤ 0.1; Figure 6B). In comparison, DMBA exposure in lean compared to obese mice resulted in alterations in 60.5% of the total ovarian proteome and 47.3% of the SUMO-ome (*P* ≤ 0.1; Figure 6B), Among the 114 proteins detected, 29 were in common with the whole ovary proteome (Supplemental Table 7). To pinpoint SUMO-2/3 substrates whose levels of SUMOylation were affected by the treatments, 20 proteins enriched or under-enriched by SUMOylation due to treatment were identified, which were detected but not altered in the whole ovary proteome (Table 4).

## Discussion

Ovarian function is foundational to female reproductive health, governing oocyte development, ovulation and hormone production [1, 55]. The finite number of primordial follicles present at birth dictates the reproductive lifespan, with their depletion leading to infertility [1]. This depletion can occur naturally via atresia or can be accelerated by various factors, including environmental exposures and lifestyle [56–58]. Genotoxic exposures, encompassing industrial chemicals, air pollutants, pesticides and radiation sources pose a threat to ovarian function [59–61]. Exposure to environmental pollutants including DMBA can negatively impact ovarian function via induction of DNA DSB [62] and SUMOs and protein inhibitor of activated STAT (a SUMO ligase enzyme E3), localize at DSB foci [53].

Obesity is a global health threat that impairs oocyte quality [63], decreases fecundity [64], increases risk of congenital disabilities [65], premature births [66] and stillbirths [67], and is associated with polycystic ovarian syndrome [68]. Obesity can diminish the ovarian follicular reserve [69], induce ovarian DNA damage [70] and elevate oxidative stress [71]. Obesity can also impact ovarian sensitivity to ovotoxicants by affecting the basal abundance and responsiveness of DDR proteins [71, 72].

A decline in primordial follicle number due to obesity [69] and DMBA [15] individually have been reported, and hyperphagia-induced obesity differentially impacts the ovarian proteomic response to DMBA-induced genotoxicity [51, 52]. Additionally, a follicle-specific abundance and DDR protein response was demonstrated, suggesting PTM involvement in regulating the impact of obesity on ovotoxicity. A protein group identified to be affected by DMBA exposure in obese mice were the SUMO proteins [52], and considering their identified roles in female fertility [48, 49], they were identified as an attractive candidate to explore.

Thus, the role of SUMOylation during DMBA-induced ovotoxicity in lean and obese mice was investigated by recapitulating the identical experimental design in ~10-week-old lean and obese mice [52]. In the ovary, 114 SUMOylated proteins were identified by LC–MS to be components of the ovarian SUMO-ome. The interactome generated by the SUMO-2/3 targets identified in the ovary exhibited high functional and physical interactions, emphasizing the biological relevance of this group of proteins. Particularly, some of the substrates could be assigned to cancer pathways (P53, angiogenesis, apoptosis signaling, cadherin signaling, cell cycle, FGF signaling, EGF receptor signaling, ubiquitin-proteasome, WNT signaling) [73, 74]. Thus, identification of ovarian protein targets of SUMOylation has relevance to understanding basic ovarian physiology and reproductive pathologies.

**Table 4:** Enriched and under-enriched SUMOylated proteins.

*Treatment*	*UNIPROT ID*	*Protein name*	*FC*	*P value*
LC vs LD	P62259	14–3-3 protein epsilon (14–3-3E)	−1.34	0.01
LC VS OC	P62889	Large ribosomal subunit protein eL30	−2.59	< 0.001
	P61205	ADP-ribosylation factor 3	−1.61	< 0.001
	P68372	Tubulin beta-4B chain	−1.57	< 0.001
	P05213	Tubulin alpha-1B chain	−1.57	< 0.001
	P11499	Heat shock protein HSP 90-beta	−1.56	0.01
	P07901	Heat shock protein HSP 90-alpha	−1.02	0.03
	P62242	40S ribosomal protein S8	−0.99	0.03
	P07356	Annexin A2	−0.85	0.03
	P51881	ADP/ATP translocase 2	−0.76	0.04
	P09103	Protein disulfide-isomerase (PDI)	1.06	0.02
	P27546	Microtubule-associated protein 4 (MAP-4)	1.84	0.01
OC vs OD	P01027	Complement C3	1.30	< 0.001
	P27546	Microtubule-associated protein 4 (MAP-4)	1.82	< 0.001
	O70456	14–3-3 protein sigma (Stratifin)	1.92	< 0.001
	Q61781	Keratin, type I cytoskeletal 14	2.35	< 0.001
LD vs OD	Q61781	Keratin, type I cytoskeletal 14	3.02	< 0.001
	P68372	Tubulin beta-4B chain	−2.89	0.01
	A8DUK4	Beta-globin	0.93	0.05
	P09103	Protein disulfide-isomerase	1.36	0.02

SUMOylation is involved in cellular tumor antigen (TP53) and E3 ubiquitin protein ligase (MDM2) interaction, inducing death of tumor cells [75], enhancing substrate binding activity of heat shock protein (HSP) 90 [76] and activating rat sarcoma virus protein [77], all of which were identified as ovarian SUMO-2/3 targets herein. Indeed, several HSPs were identified as targets of SUMO2/3 including HSPA1L, HSPA8, and HSP90 and there is evidence for HSP to be involved in ovarian pathology including obesity-related ovarian cancer [78] and polycystic ovary syndrome [79]. SUMOylation is involved in nucleocytoplasmic transport, regulating the movement of proteins involved in several cellular processes, including transcriptional regulation [80], DDR [81], chromatin remodeling [82] and apoptosis [83]. There is emerging evidence for SUMOylation’s role in controlling the structure of cytoskeleton proteins [84] which was the largest category of ovarian proteins identified in this study. Cytoskeleton proteins have essential roles in spindle assembly and spindle migration during oocyte maturation [85, 86] and dramatic changes to cytoskeletal proteins are observed in the granulosa cells of cystic follicles [87], emphasizing their role in proper ovarian functioning. Furthermore, SUMOylation is essential for RNA trafficking between the nucleus and cytoplasm [88] and several translational proteins (ribosomal subunits, elongation factor, eukaryotic translational initiation factor 2 subunit three and 26S proteasome component) were identified as ovarian SUMO-2/3 targets in this study. These findings support that SUMOylation is important in ovarian function and identify the ovarian SUMO-ome.

To investigate the influence of obesity and DMBA exposure, individually and combined, on the ovarian SUMO-ome, SUMO-2/3 targets were compared between lean and obese mice treated with vehicle control or DMBA. In lean mice, DMBA exposure altered 18 proteins and tended to alter 3 proteins, one of which was also identified in a whole ovary proteomic profile [52]: 14–3-3 protein epsilon. In obese mice, DMBA altered 29 proteins with a tendency for an additional seven to be changed, several of which were identified in the proteomic changes to the whole ovary [52]. Three proteins were identified with chromatin-related roles; large proline-rich protein BAG6, which functions in DNA damage-induced apoptosis [89], bromodomain-containing protein 1, which is a scaffold subunit of histone acetyltransferases [90], and importin, which mediates nuclear transport of DNA repair proteins [91]; that were decreased by DMBA exposure in lean mice but increased in DMBA-exposed lean when compared to obese mice. This suggests a differential epigenetic control of the DDR due to metabolic status. Proteins that were altered by DMBA regardless of metabolic status, were all decreased. There are few literature reports about SUMOylation modification of five of these proteins but one, nucleolar protein 56 (NOP56), is reported as a weak SUMO target [92].

Obesity alone affected the SUMOylation profile of 55 proteins with a tendency for an additional 11 proteins to be changed, and there is indeed some evidence of the regulatory actions of SUMOylation during the metabolic syndrome. For example, SUMO-1 regulates peroxisomal proliferator-activated receptor γ, which is essential for the development of adipocytes [93], thyroid hormone receptor SUMOylation is required during preadipocyte proliferation [94] and ablation of SUMOylation causes irregular [95] or reduced adipose lipid storage [96]. Exposure of obese mice to DMBA affected 35 SUMO-target proteins. Interestingly, the directionality of these changes was not influenced by DMBA treatment, indicating that obesity has a more dominant role in the modulation of SUMOylation than DMBA exposure alone. This was not a surprising observation since obesity is a state of low-grade, chronic inflammation that creates a stressful environment at the cellular level [97]. To narrow down the obesity-influenced SUMO-2/3 targets, this list was compared against whole ovary proteomic changes in response to obesity [52] and nine protein targets were identified. These proteins are involved in cell cycle regulation, cytoskeletal structure and translation and are potential candidates for studying modifications by SUMO-2/3 due to obesity during genotoxicant exposure. SUMOylation is being explored as a target for developing novel anti-diabetic drugs [98, 99] and proteins identified in this study could aid in such explorations.

Approximately one-third of all proteins carry a PTM [100] and the SUMO-ome detected by SUMO-2/3 was ~9% of the total number of proteins detected in the whole ovary proteome. Of these, in lean mice, DMBA exposure altered SUMOylation of the least number of proteins, ~11%, whereas obesity altered the most, ~58%, among treatment comparisons. This suggests that obesity alone has a profound impact on the SUMOylation profile of the ovarian proteome, and that metabolic status is an important factor in the alteration of PTM in the context of ovarian toxicology. Interestingly, SAE1 was decreased in obese mice, likely because SAE1 dimerizes with SAE2 to activate SUMO proteins, and all three mouse SUMO isoforms were increased in DMBA-exposed obese mice in the total ovarian proteome [52] corresponding to the increased levels of SUMO activity observed in these treatments. Thus, a SUMO-2/3 antibody was utilized to precipitate SUMO-2/3 targets. It would also be useful to identify targets of SUMO-1 modification since the abundance of SUMO-1 was also increased in obese mice exposed to DMBA [52], providing a holistic profile of ovarian SUMOylation. It is also noteworthy that there were several proteins identified as SUMO substrates in this experiment that were not identified in the whole ovary in the previous study [52]. Nevertheless, the SUMO substrates identified, with stringent selection criteria, are strong candidates for further exploration and are foundational toward understanding roles of SUMO proteins during ovarian function. The impact of oocyte-specific *Ube2i* loss has recently identified metabolism-related proteins as SUMO targets and noted that the affected proteins did not differ transcriptionally, further supporting SUMOylation as important in the ovary for protein-level regulation [101].

In conclusion, this study identified ovarian SUMO-2/3 targets and elucidated the impact of obesity and DMBA exposure on the ovarian SUMO-ome, revealing alterations in protein SUMOylation profiles. The findings suggest that obesity, independent of DMBA exposure, influences the SUMOylation of several ovarian proteins involved in critical biological processes. Furthermore, the findings support that DMBA exposure selectively affects the SUMOylation of a subset of proteins, providing insights into the ovotoxic molecular mechanisms of obesity and chemical exposure in ovarian function. Obesity may perturb ovarian homeostasis by modulating the PTM landscape and SUMOylation may serve as a regulatory mechanism affecting key cellular processes employed by ovarian cells to respond to ovotoxic stress in both lean and obese mice. Investigations to predict and identify specific protein SUMOylation sites are warranted, and the data herein could be utilized in predicting and testing the specific SUMOylation sites to further elucidate the role of SUMOylation during ovotoxic stress. Further, elucidating the exact ovarian geographical location of SUMO-target proteins and deciphering if there is an upstream SUMOylation regulator differing between lean and obese mice would have value.

## Supplementary Material

Supplemental_Figure_1_ioaf101

Supplemental_Table_1_ioaf101

Supplemental_Table_2_ioaf101

Supplemental_Table_3_ioaf101

Supplemental_Table_4_ioaf101

Supplemental_Table_5_ioaf101

Supplemental_Table_6_ioaf101

Supplemental_Table_7_ioaf101

## Data Availability

Data available on reasonable request to the senior author.
