# Supplementary material for: Ovarian SUMO-2/3 targets and their differential response to genotoxic stress induced by 7,12-dimethylbenz(a) anthracene exposure in lean and obese female mice
Source: Biol Reprod. 2025 Apr 30;113(4):962–76. doi: 10.1093/biolre/ioaf101 (PMC12527294; doi:10.1093/biolre/ioaf101)
Supplement: Supplemental_Figure_1_ioaf101 [file supplemental_figure_1_ioaf101.docx]

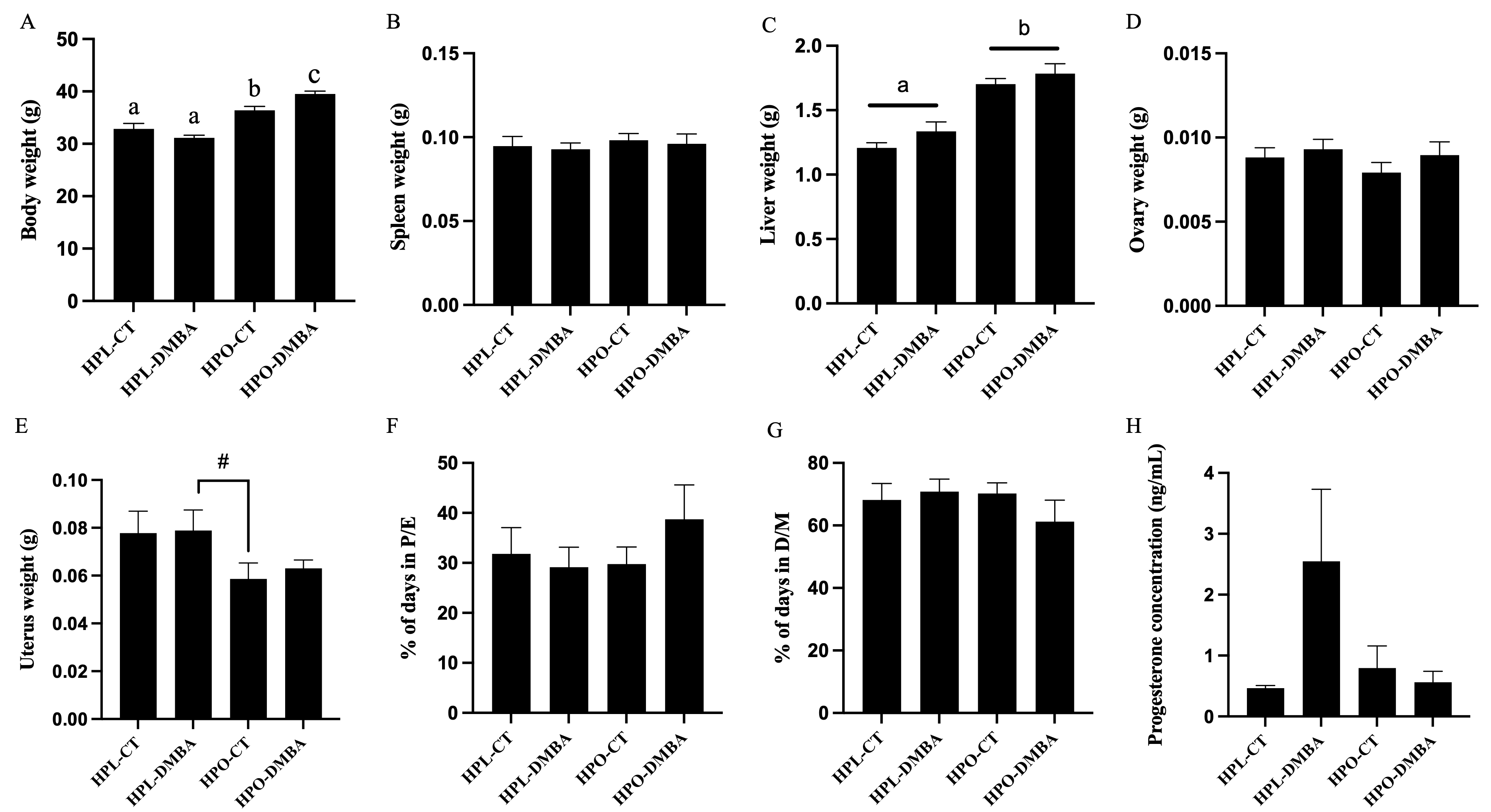


**Supplemental Figure 1. Impact of DMBA exposure on total body and organ weight in lean and hyperphagia-induced obese mice**. Following 7 d of exposure to corn oil (CT) or DMBA in lean (HPL) or obese (HPO) mice, weights of **(A)** body, **(B)** spleen, **(C)** liver, **(D)** ovary, and **(E**) uterus were recorded. The total percentage of days spent in **(F)** proestrus + estrus and **(G)** diestrus + metestrus. **(H)** The amount of circulating serum progesterone was measured via ELISA. Different letters indicate differences between treatments; P < 0.05; ^#^P < 0.1; n = 4-6 for progesterone concentration; n = 10 for the rest of the endpoints.
