# Supplementary material for: Ovarian SUMO-2/3 targets and their differential response to genotoxic stress induced by 7,12-dimethylbenz(a) anthracene exposure in lean and obese female mice
Source: Biol Reprod. 2025 Apr 30;113(4):962–76. doi: 10.1093/biolre/ioaf101 (PMC12527294; doi:10.1093/biolre/ioaf101)
Supplement: Supplemental_Table_1_ioaf101 [file supplemental_table_1_ioaf101.docx]

**Supplemental Table 1.** String Network Analysis of functional interactions of SUMOylated proteins.

| **Functional Interactions String Network Statistics** | |
| --- | --- |
| Number of Nodes | 103 |
| Number of Edges | 523 |
| Average Node Degree | 10.2 |
| Average Local Clustering Coefficient | 0.454 |
| Expected Number of Edges | 228 |
| PPI enrichment p-value | < 1.0 e^-16^ |
