# Supplementary material for: Ovarian SUMO-2/3 targets and their differential response to genotoxic stress induced by 7,12-dimethylbenz(a) anthracene exposure in lean and obese female mice
Source: Biol Reprod. 2025 Apr 30;113(4):962–76. doi: 10.1093/biolre/ioaf101 (PMC12527294; doi:10.1093/biolre/ioaf101)
Supplement: Supplemental_Table_2_ioaf101 [file supplemental_table_2_ioaf101.docx]

**Supplemental Table 2.** String Network Analysis of physical interactions of SUMOylated proteins.

| **Physical Interactions String Network Statistics** | |
| --- | --- |
| Number of Nodes | 103 |
| Number of Edges | 113 |
| Average Node Degree | 2.29 |
| Average Local Clustering Coefficient | 0.415 |
| Expected Number of Edges | 53 |
| PPI enrichment p-value | 7.11 e^-15^ |
