# Supplementary material for: Ovarian SUMO-2/3 targets and their differential response to genotoxic stress induced by 7,12-dimethylbenz(a) anthracene exposure in lean and obese female mice
Source: Biol Reprod. 2025 Apr 30;113(4):962–76. doi: 10.1093/biolre/ioaf101 (PMC12527294; doi:10.1093/biolre/ioaf101)
Supplement: Supplemental_Table_3_ioaf101 [file supplemental_table_3_ioaf101.docx]

**Supplemental Table 3**. SUMOylated ovarian proteins altered by obesity in mice.

| UNIPROT ID | Protein Name |  | Log2(FC) | *P* value | FDR |
| --- | --- | --- | --- | --- | --- |
| A0A075B5V6 | **Immunoglobulin heavy variable V1-42** |  | 3.73 | <0.0001 | <0.001 |
| B1ARW8 | **Uncharacterized protein C1orf122 homolog** |  | -3.33 | <0.0001 | <0.001 |
| Q9Z0N1 | **Eukaryotic translation initiation factor 2 subunit 3, X-linked** |  | -2.44 | <0.0001 | <0.001 |
| Q80U49 | **Centrosomal protein of 170 kDa protein B** |  | -3.56 | <0.0001 | <0.001 |
| Q9CXD6 | **Mitochondrial calcium uniporter regulator 1** |  | -2.87 | <0.0001 | <0.001 |
| P60843 | **Eukaryotic initiation factor 4A-I** |  | -2.78 | <0.0001 | <0.001 |
| Q6R0H6 | **Protein ALEX** |  | -2.87 | <0.0001 | <0.001 |
| Q8BFZ3 | **Beta-actin-like protein 2** |  | -2.10 | <0.0001 | <0.001 |
| P62889 | **Large ribosomal subunit protein eL30** |  | -2.59 | <0.0001 | <0.001 |
| A0A140T8M4 | **Immunoglobulin kappa variable 8-19** |  | 2.11 | <0.0001 | <0.001 |
| Q8R0F5 | **RNA-binding motif protein, X-linked 2** |  | -2.33 | <0.0001 | <0.001 |
| A0A140LJ72 | **Mucin 16** |  | -3.70 | <0.0001 | <0.001 |
| O88942 | **Nuclear factor of activated T-cells, cytoplasmic 1** |  | -1.70 | <0.0001 | <0.001 |
| P05213 | **Tubulin alpha-1B chain** |  | -1.94 | <0.0001 | <0.001 |
| A0A0A0MQC1 | **Immunoglobulin heavy variable 3-5** |  | 2.62 | <0.0001 | <0.001 |
| Q61315 | **Adenomatous polyposis coli protein** |  | -1.78 | 8.82E-04 | 1.58E-03 |
| P61205 | **ADP-ribosylation factor 3** |  | -1.61 | 8.82E-04 | 1.58E-03 |
| Q80U72 | **Protein scribble homolog** |  | -1.90 | 8.82E-04 | 1.58E-03 |
| P01865 | **Ig gamma-2A chain C region, membrane-bound form** |  | 2.35 | 8.82E-04 | 1.58E-03 |
| O88502 | **High affinity cAMP-specific and IBMX-insensitive 3',5'-cyclic phosphodiesterase 8A** |  | 1.51 | 5.00E-03 | 0.01 |
| P24529 | **Tyrosine 3-monooxygenase** |  | -1.25 | 5.00E-03 | 0.01 |
| P68372 | **Tubulin beta-4B chain** |  | -1.57 | 5.00E-03 | 0.01 |
| P97350 | **Plakophilin-1** |  | 1.48 | 0.01 | 0.02 |
| P11499 | **Heat shock protein HSP 90-beta** |  | -1.56 | 0.01 | 0.02 |
| P53994 | **Ras-related protein Rab-2A** |  | -1.25 | 0.01 | 0.02 |
| P27546 | **Microtubule-associated protein 4** |  | 1.84 | 0.01 | 0.02 |
| P62192 | **26S proteasome regulatory subunit 4** |  | -0.93 | 0.02 | 0.03 |
| P42859 | **Huntingtin** |  | 1.05 | 0.02 | 0.03 |
| E9QLU9 | **FYVE, RhoGEF and PH domain containing 5** |  | -1.45 | 0.02 | 0.03 |
| Q925H7 | **Keratin-associated protein 19-4** |  | -1.20 | 0.02 | 0.03 |
| P09103 | **Protein disulfide-isomerase** |  | 1.06 | 0.02 | 0.03 |
| P62242 | **Small ribosomal subunit protein eS8** |  | -0.99 | 0.03 | 0.03 |
| P07356 | **Annexin A2** |  | -0.85 | 0.03 | 0.03 |
| Q9DCR2 | **AP-3 complex subunit sigma-1** |  | 1.00 | 0.03 | 0.03 |
| Q8BIK4 | **Dedicator of cytokinesis protein 9** |  | 1.09 | 0.03 | 0.03 |
| G5E8P1 | **Bromodomain-containing protein 1** |  | 1.20 | 0.03 | 0.03 |
| Q5XG71 | **Small subunit processome component 20 homolog** |  | 1.03 | 0.03 | 0.03 |
| P62259 | **14-3-3 protein epsilon** |  | 1.07 | 0.03 | 0.03 |
| A0A075B5P4 | Immunoglobulin heavy constant gamme 1 (G1m marker) |  | 1.55 | 0.03 | 0.03 |
| Q5RKT9 | **Mannoside acetylglucosaminyltransferase 3** |  | 1.08 | 0.03 | 0.03 |
| P07901 | **Heat shock protein HSP 90-alpha** |  | -1.02 | 0.03 | 0.03 |
| P01942 | **Hemoglobin subunit alpha** |  | 1.03 | 0.03 | 0.03 |
| Q9WTM5 | **RuvB-like 2** |  | 0.96 | 0.04 | 0.03 |
| P62827 | **GTP-binding nuclear protein Ran** |  | -0.73 | 0.04 | 0.03 |
| Q3V2K7 | **Nucleoporin 50 like** |  | -0.59 | 0.04 | 0.03 |
| P62301 | **Small ribosomal subunit protein uS15** |  | -0.89 | 0.04 | 0.03 |
| Q80T14 | **Extracellular matrix organizing protein FRAS1** |  | -0.80 | 0.04 | 0.03 |
| Q02257 | **Junction plakoglobin** |  | 1.06 | 0.04 | 0.03 |
| A0A286YDM5 | **Olfactory receptor** |  | 1.09 | 0.04 | 0.03 |
| Q9D6Z1 | **Nucleolar protein 56** |  | 0.83 | 0.04 | 0.03 |
| Q8VI75 | **Importin-4** |  | 1.00 | 0.04 | 0.03 |
| Q3UVL4 | **Vacuolar protein sorting-associated protein 51 homolog** |  | -0.70 | 0.04 | 0.03 |
| Q9Z1R2 | **Large proline-rich protein BAG6** |  | 0.84 | 0.04 | 0.03 |
| P62858 | **Small ribosomal subunit protein eS28** |  | -0.71 | 0.04 | 0.03 |
| P51881 | **ADP/ATP translocase 2** |  | -0.76 | 0.04 | 0.03 |
| Q9D708 | **Protein S100-A16** |  | 0.94 | 0.06 | 0.03 |
| P68134 | **Actin, alpha skeletal muscle** |  | 0.78 | 0.06 | 0.03 |
| Q64467 | **Glyceraldehyde-3-phosphate dehydrogenase, testis-specific** |  | 0.80 | 0.06 | 0.03 |
| P62264 | **Small ribosomal subunit protein uS11** |  | 0.77 | 0.06 | 0.03 |
| P35279 | **Ras-related protein Rab-6A** |  | -0.64 | 0.07 | 0.04 |
| Q9JI91 | **Alpha-actinin-2** |  | -0.57 | 0.07 | 0.04 |
| P54071 | **Isocitrate dehydrogenase [NADP], mitochondrial** |  | 0.77 | 0.07 | 0.04 |
| P51150 | **Ras-related protein Rab-7a** |  | -0.59 | 0.08 | 0.04 |
| Q99PV0 | **Pre-mRNA-processing-splicing factor 8** |  | -0.50 | 0.09 | 0.05 |
| O70456 | **14-3-3 protein sigma** |  | 0.59 | 0.09 | 0.05 |
| Q921I1 | **Serotransferrin** |  | 0.61 | 0.10 | 0.05 |
