# Supplementary material for: Ovarian SUMO-2/3 targets and their differential response to genotoxic stress induced by 7,12-dimethylbenz(a) anthracene exposure in lean and obese female mice
Source: Biol Reprod. 2025 Apr 30;113(4):962–76. doi: 10.1093/biolre/ioaf101 (PMC12527294; doi:10.1093/biolre/ioaf101)
Supplement: Supplemental_Table_4_ioaf101 [file supplemental_table_4_ioaf101.docx]

**Supplemental Table 4**. SUMOylated ovarian proteins altered by DMBA exposure in lean mice.

| UNIPROT ID | Protein Name | Log2(FC) | | | *P* value | FDR |
| --- | --- | --- | --- | --- | --- | --- |
| Q9DCR2 | **AP-3 complex subunit sigma-1** | | -4.07 | | <0.0001 | <0.001 |
| Q5XG71 | **Small subunit processome component 20 homolog** | | | -3.78 | <0.0001 | <0.001 |
| Q8BIK4 | **Dedicator of cytokinesis protein 9** | | | -3.03 | <0.0001 | <0.001 |
| Q9Z1R2 | **Large proline-rich protein BAG6** | | | -1.74 | <0.0001 | <0.001 |
| G5E8P1 | **Bromodomain-containing protein 1** | | | -2.56 | <0.0001 | <0.001 |
| Q8VI75 | **Importin-4** | | | -1.80 | <0.0001 | <0.001 |
| O88502 | **High affinity cAMP-specific and IBMX-insensitive 3',5'-cyclic phosphodiesterase 8A** | | | -2.16 | 6.67E-04 | 0.01 |
| A0A140T8M4 | **Immunoglobulin kappa variable 8-19** | | | -1.81 | 1.11E-03 | 0.01 |
| P42859 | **Huntingtin** | | | -1.25 | 2.00E-03 | 0.02 |
| Q5RKT9 | **Mannoside acetylglucosaminyltransferase 3** | | | -1.91 | 3.11E-03 | 0.03 |
| Q9D6Z1 | **Nucleolar protein 56** | | | -1.32 | 4.11E-03 | 0.03 |
| P97350 | **Plakophilin-1** | | | 1.81 | 4.78E-03 | 0.04 |
| P62259 | **14-3-3 protein epsilon** | | | -1.34 | 0.01 | 0.04 |
| E9Q557 | **Desmoplakin** | | | 1.61 | 0.02 | 0.16 |
| P50247 | **Adenosylhomocysteinase** | | | 1.11 | 0.03 | 0.19 |
| Q61781 | **Keratin, type I cytoskeletal 14** | | | 1.17 | 0.05 | 0.26 |
| P16627 | **Heat shock 70 kDa protein 1-like** | | | 1.14 | 0.05 | 0.26 |
| Q925H7 | **Keratin-associated protein 19-4** | | | -0.85 | 0.05 | 0.26 |
| P10126 | **Elongation factor 1-alpha 1** | | | 0.95 | 0.06 | 0.27 |
| A0A075B5P5 | **Immunoglobulin heavy constant gamma 3** | | | -0.93 | 0.09 | 0.38 |
| Q8BFZ3 | **Beta-actin-like protein 2** | | | -0.63 | 0.09 | 0.40 |
