# Supplementary material for: Ovarian SUMO-2/3 targets and their differential response to genotoxic stress induced by 7,12-dimethylbenz(a) anthracene exposure in lean and obese female mice
Source: Biol Reprod. 2025 Apr 30;113(4):962–76. doi: 10.1093/biolre/ioaf101 (PMC12527294; doi:10.1093/biolre/ioaf101)
Supplement: Supplemental_Table_5_ioaf101 [file supplemental_table_5_ioaf101.docx]

**Supplemental Table 5**. SUMOylated ovarian proteins altered by DMBA exposure in obese mice.

| UNIPROT ID | Protein Name | Log2(FC) | *P* value | | | FDR |
| --- | --- | --- | --- | --- | --- | --- |
| P60710 | **Actin, cytoplasmic 1** | 1.06 | | <0.0001 | <0.001 | |
| Q61781 | **Keratin, type I cytoskeletal 14** | 3.02 | | <0.0002 | <0.002 | |
| P27546 | **Microtubule-associated protein 4** | 1.82 | | <0.0003 | <0.003 | |
| Q9DCR2 | **AP-3 complex subunit sigma-1** | -4.41 | | <0.0004 | <0.004 | |
| P05977 | **Myosin light chain 1/3, skeletal muscle isoform** | 2.35 | | <0.0005 | <0.005 | |
| P13541 | **Myosin-3** | 2.77 | | <0.0006 | <0.006 | |
| Q80W71 | **Pleckstrin homology domain-containing family A member 8** | 2.09 | | <0.0007 | <0.007 | |
| Q02566 | **Myosin-6** | 2.04 | | 5.56E-04 | 4.00E-03 | |
| E9Q557 | **Desmoplakin** | 1.61 | | 5.56E-04 | 4.00E-03 | |
| P42859 | **Huntingtin** | -2.39 | | 8.33E-04 | 4.00E-03 | |
| P07310 | **Creatine kinase M-type** | 1.34 | | 8.33E-04 | 4.00E-03 | |
| P01027 | **Complement C3** | 1.30 | | 8.33E-04 | 4.00E-03 | |
| A0A075B5P4 | **Immunoglobulin heavy constant gamma 1 (G1m marker)** | 1.40 | | 8.33E-04 | 4.00E-03 | |
| O70456 | **14-3-3 protein sigma** | 1.92 | | 3.89E-03 | 0.02 | |
| P58252 | **Elongation factor 2** | -2.61 | | 4.72E-03 | 0.02 | |
| P16858 | **Glyceraldehyde-3-phosphate dehydrogenase** | 0.83 | | 4.72E-03 | 0.02 | |
| Q5XG71 | **Small subunit processome component 20 homolog** | -2.60 | | 4.72E-03 | 0.02 | |
| P97457 | **Myosin regulatory light chain 11** | 1.41 | | 4.72E-03 | 0.02 | |
| Q8BIK4 | **Dedicator of cytokinesis protein 9** | -3.06 | | 4.72E-03 | 0.02 | |
| A0A0A0MQC1 | **Immunoglobulin heavy variable 3-5** | 0.78 | | 0.01 | 0.02 | |
| P05064 | **Fructose-bisphosphate aldolase A** | 0.91 | | 0.01 | 0.02 | |
| O88502 | **High affinity cAMP-specific and IBMX-insensitive 3',5'-cyclic phosphodiesterase 8A** | -2.30 | | 0.01 | 0.04 | |
| P68134 | **Actin, alpha skeletal muscle** | 0.61 | | 0.02 | 0.04 | |
| O88990 | **Alpha-actinin-3** | 0.78 | | 0.02 | 0.05 | |
| Q3UVL4 | **Vacuolar protein sorting-associated protein 51 homolog** | 0.51 | | 0.03 | 0.07 | |
| A8DUK4 | **Beta-globin** | 0.58 | | 0.03 | 0.08 | |
| Q8R0F5 | **RNA-binding motif protein, X-linked 2** | -1.47 | | 0.03 | 0.08 | |
| Q9D6Z1 | **Nucleolar protein 56** | -1.82 | | 0.04 | 0.09 | |
| Q921I1 | **Serotransferrin** | 0.44 | | 0.04 | 0.09 | |
| A0A075B5V6 | **Immunoglobulin heavy variable V1-42** | -0.40 | | 0.06 | 0.13 | |
| P08113 | **Endoplasmin** | 0.24 | | 0.07 | 0.13 | |
| Q02257 | **Junction plakoglobin** | 0.11 | | 0.07 | 0.13 | |
| P16627 | **Heat shock 70 kDa protein 1-like** | -1.52 | | 0.07 | 0.14 | |
| Q5RKT9 | Mannoside acetylglucosaminyltransferase 3 | -1.65 | | 0.08 | 0.14 | |
| P53994 | **Ras-related protein Rab-2A** | -0.95 | | 0.10 | 0.17 | |
| Q99PV0 | **Pre-mRNA-processing-splicing factor 8** | 0.56 | | 0.10 | 0.17 | |
