# Supplementary material for: Ovarian SUMO-2/3 targets and their differential response to genotoxic stress induced by 7,12-dimethylbenz(a) anthracene exposure in lean and obese female mice
Source: Biol Reprod. 2025 Apr 30;113(4):962–76. doi: 10.1093/biolre/ioaf101 (PMC12527294; doi:10.1093/biolre/ioaf101)
Supplement: Supplemental_Table_6_ioaf101 [file supplemental_table_6_ioaf101.docx]

**Supplemental Table 6**. SUMOylated proteins altered in lean compared to obese mice identically exposed to DMBA.

| UNIPROT ID | Protein Name | Log2FC | *P* value | FDR |
| --- | --- | --- | --- | --- |
| P27546 | **Microtubule-associated protein 4** | 3.62 | <0.0001 | <0.001 |
| Q5XG71 | **Small subunit processome component 20 homolog** | 2.20 | <0.0001 | <0.001 |
| P60710 | **Actin, cytoplasmic 1** | 0.90 | <0.0001 | <0.001 |
| A0A075B5V6 | **Immunoglobulin heavy variable V1-42** | 3.03 | <0.0001 | <0.001 |
| A0A140T8M4 | **Immunoglobulin kappa variable 8-19** | 3.58 | <0.0001 | <0.001 |
| Q9Z1R2 | **Large proline-rich protein BAG6** | 1.93 | 8.62E-04 | 5.00E-03 |
| Q80W71 | **Pleckstrin homology domain-containing family A member 8** | 2.15 | 2.76E-03 | 0.01 |
| A0A0A0MQC1 | **Immunoglobulin heavy variable 3-5** | 3.25 | 3.97E-03 | 0.01 |
| A0A075B5P4 | **Immunoglobulin heavy constant gamma 1 (G1m marker)** | 2.98 | 3.97E-03 | 0.01 |
| P16858 | **Glyceraldehyde-3-phosphate dehydrogenase** | 1.31 | 4.31E-03 | 0.01 |
| P53994 | **Ras-related protein Rab-2A** | -2.24 | 4.83E-03 | 0.01 |
| Q8R0F5 | **RNA-binding motif protein, X-linked 2** | -3.43 | 4.83E-03 | 0.01 |
| Q8VI75 | **Importin-4** | 1.58 | 4.83E-03 | 0.01 |
| Q61781 | **Keratin, type I cytoskeletal 14** | 2.35 | 4.83E-03 | 0.01 |
| P05977 | **Myosin light chain 1/3, skeletal muscle isoform** | 2.41 | 4.83E-03 | 0.01 |
| Q02566 | **Myosin-6** | 2.25 | 0.01 | 0.01 |
| O70456 | **14-3-3 protein sigma** | 2.96 | 0.01 | 0.01 |
| O88502 | **High affinity cAMP-specific and IBMX-insensitive 3',5'-cyclic phosphodiesterase 8A** | 1.38 | 0.01 | 0.01 |
| P07310 | **Creatine kinase M-type** | 1.05 | 0.01 | 0.01 |
| P68134 | **Actin, alpha skeletal muscle** | 1.51 | 0.01 | 0.01 |
| P58252 | **Elongation factor 2** | -2.63 | 0.01 | 0.01 |
| P13541 | **Myosin-3** | 2.57 | 0.01 | 0.01 |
| O88942 | **Nuclear factor of activated T-cells, cytoplasmic 1** | -1.89 | 0.01 | 0.01 |
| Q02257 | **Junction plakoglobin** | 1.29 | 0.01 | 0.01 |
| E9Q557 | **Desmoplakin** | 0.70 | 0.01 | 0.01 |
| P68372 | **Tubulin beta-4B chain** | -2.89 | 0.01 | 0.02 |
| P01027 | **Complement C3** | 1.59 | 0.01 | 0.02 |
| P62259 | **14-3-3 protein epsilon** | 0.95 | 0.01 | 0.02 |
| Q80U49 | **Centrosomal protein of 170 kDa protein B** | -3.59 | 0.01 | 0.02 |
| P09103 | **Protein disulfide-isomerase** | 1.36 | 0.02 | 0.03 |
| P60843 | **Eukaryotic initiation factor 4A-I** | -3.04 | 0.02 | 0.03 |
| Q5RKT9 | **Mannoside acetylglucosaminyltransferase 3** | 1.35 | 0.02 | 0.03 |
| Q8BIK4 | **Dedicator of cytokinesis protein 9** | 1.06 | 0.02 | 0.03 |
| B1ARW8 | **Uncharacterized protein C1orf122 homolog** | -3.15 | 0.02 | 0.03 |
| Q921I1 | **Serotransferrin** | 0.67 | 0.02 | 0.04 |
| Q8VIJ8 | **GATOR1 complex protein NPRL3** | 0.58 | 0.03 | 0.04 |
| P62889 | **Large ribosomal subunit protein eL30** | -2.98 | 0.03 | 0.04 |
| P05064 | **Fructose-bisphosphate aldolase A** | 0.88 | 0.04 | 0.05 |
| P62301 | **Small ribosomal subunit protein uS15** | -2.13 | 0.05 | 0.06 |
| G5E8P1 | **Bromodomain-containing protein 1** | 2.40 | 0.05 | 0.06 |
| P61205 | **ADP-ribosylation factor 3** | -1.70 | 0.05 | 0.06 |
| A8DUK4 | **Beta-globin** | 0.93 | 0.05 | 0.06 |
| P01942 | **Hemoglobin subunit alpha** | 0.42 | 0.05 | 0.06 |
| P97457 | **Myosin regulatory light chain 11** | 1.00 | 0.08 | 0.09 |
| Q9CZB9 | **Transmembrane protein 128** | 0.68 | 0.09 | 0.10 |
| P16627 | **Heat shock 70 kDa protein 1-like** | -2.32 | 0.09 | 0.10 |
| O88990 | **Alpha-actinin-3** | 0.35 | 0.09 | 0.10 |
