# Supplementary material for: Ovarian SUMO-2/3 targets and their differential response to genotoxic stress induced by 7,12-dimethylbenz(a) anthracene exposure in lean and obese female mice
Source: Biol Reprod. 2025 Apr 30;113(4):962–76. doi: 10.1093/biolre/ioaf101 (PMC12527294; doi:10.1093/biolre/ioaf101)
Supplement: Supplemental_Table_7_ioaf101 [file supplemental_table_7_ioaf101.docx]

**Supplemental Table 7**. SUMOylated ovarian proteins identified in the whole ovary.

| **UNIPROT** | **Protein Name** | **Protein Symbol** |
| --- | --- | --- |
| P27546 | Microtubule-associated protein 4 | MAP4 |
| P05064 | Fructose-bisphosphate aldolase A | ALDOA |
| P52480 | Pyruvate kinase PKM | PKM |
| P62889 | Large ribosomal subunit protein eL30 | RPL30 |
| A8DUK4 | Beta-globin | HBB-BS |
| P07356 | Annexin A2 | ANXA2 |
| Q03265 | ATP synthase subunit alpha, mitochondrial | ATP5F1A |
| Q8BQ46 | TAF15 RNA polymerase II, TATA box binding protein-associated factor | TAF15 |
| Q921I1 | Serotransferrin | TF |
| O70456 | 14-3-3 protein sigma | SFN |
| P01027 | Complement C3 | C3 |
| P05213 | Tubulin alpha-1B chain | TUBA1B |
| P07901 | Heat shock protein HSP 90-alpha | HSP90AA1 |
| P09103 | Protein disulfide-isomerase | P4HB |
| P10126 | Elongation factor 1-alpha 1 | EEF1A1 |
| P11499 | Heat shock protein HSP 90-beta | HSP90AB1 |
| P51881 | ADP/ATP translocase 2 | SLC25A5 |
| P50247 | Adenosylhomocysteinase | AHCY |
| P54071 | Isocitrate dehydrogenase [NADP], mitochondrial | IDH2 |
| P56480 | ATP synthase subunit beta, mitochondrial | ATP5F1B |
| P61205 | ADP-ribosylation factor 3 | ARF3 |
| P62242 | Small ribosomal subunit protein eS8 | RPS8 |
| P62259 | 14-3-3 protein epsilon | YWHAE |
| P62806 | Histone H4 | H4 |
| P62814 | V-type proton ATPase subunit B, brain isoform | ATP6V1B2 |
| P68372 | Tubulin beta-4B chain | TUBB4B |
| Q01853 | Transitional endoplasmic reticulum ATPase | VCP |
| Q61781 | Keratin, type I cytoskeletal 14 | KRT14 |
| Q6NSR8 | Probable aminopeptidase NPEPL1 | NPEPL1 |
